# Supplementary material for: Quantitative imaging of transcription in living Drosophila embryos reveals the impact of core promoter motifs on promoter state dynamics
Source: Nat Commun. 2021 Jul 23;12:4504. doi: 10.1038/s41467-021-24461-6 (PMC8302612; doi:10.1038/s41467-021-24461-6)
Supplement: Supplementary file 1 — Supplementary Information [file 41467_2021_24461_MOESM1_ESM.pdf]

Supplementary Figure 1

Gene	CAGE (Hoskins *et al.*, 2011)	RNA (Lecuyer et al., 2007; Wilk et al., 2016)	TBP (Wang *et al.*, 2014)	Sequence<br>TBP Binding Site INR TSS	Start Position (dm6)
snail (*sna*)	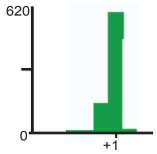	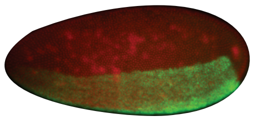	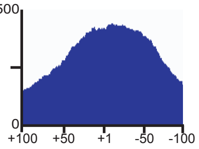	GACAGCGGCGTCGGCAGAGGCGCAGAGTTCCG<br>GG TATAAAA GAGCGTGCTCGACTGTTGACCTGTC<br>ACAGCCACCTCAGCTCTCGTTGAGAACGCAACCA	2L(-): 15,478,253
Kruppel (*kr*)	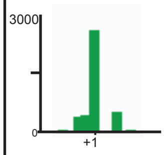	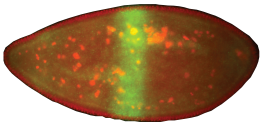	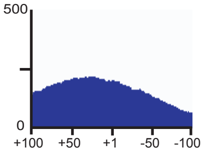	TTCGCCGAGACAGAGCGTACT TATAGTT AGCTCAC<br>GCAGCGAATTTGTATCAGTCGTGATTTGGCTCTGT<br>CAGCGAAAGGAACAACCATTTGTTGTGCC	2R(+): 21,114,141
Insulin-like peptide 4 (*Ilp4*)	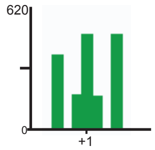	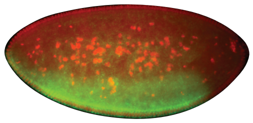	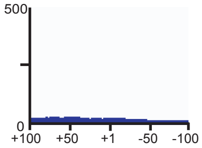	AAAGTCAATTAGCGAGTCAACATTTTGAGCGCCG<br>GCCAACTCCAAGGATCAGTATCATTTGGCATGCC<br>CAGCGATCGGTTTGCCAAGAGCACGAGAAGTT	3L(-): 9,797,353
brinker (*brk*)	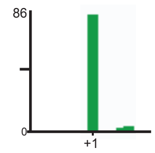	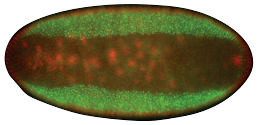	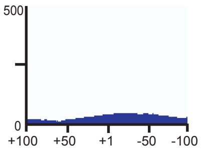	AGGCGAGGCAGTCTAGAACGTATCTTCGAACTAG<br>AACGCCGAGGGCAGAACA ATTGTGTTTTGGATTC<br>CTTGCCGTGCGGATCGCAAACGCGAGTTATGA	X(+): 7,201,976

Supplementary Figure 1: Summary of *sna*, *kr*, *Ilp4* and *brk* promoters
CAGE profile (ref. 54), representative nc14 mRNA expression pattern (ref. 52,53), TBP binding profile (ref. 84), annotated promoter sequence indicating TBP binding site (blue), Initiator element (INR, orange), and transcriptional start site (TSS, green), and genomic coordinates for the developmental promoters used in this study. For each of the four developmental promoters, the +1 position in the CAGE profile indicates the most used transcription initiation site.

Supplementary Figure 2

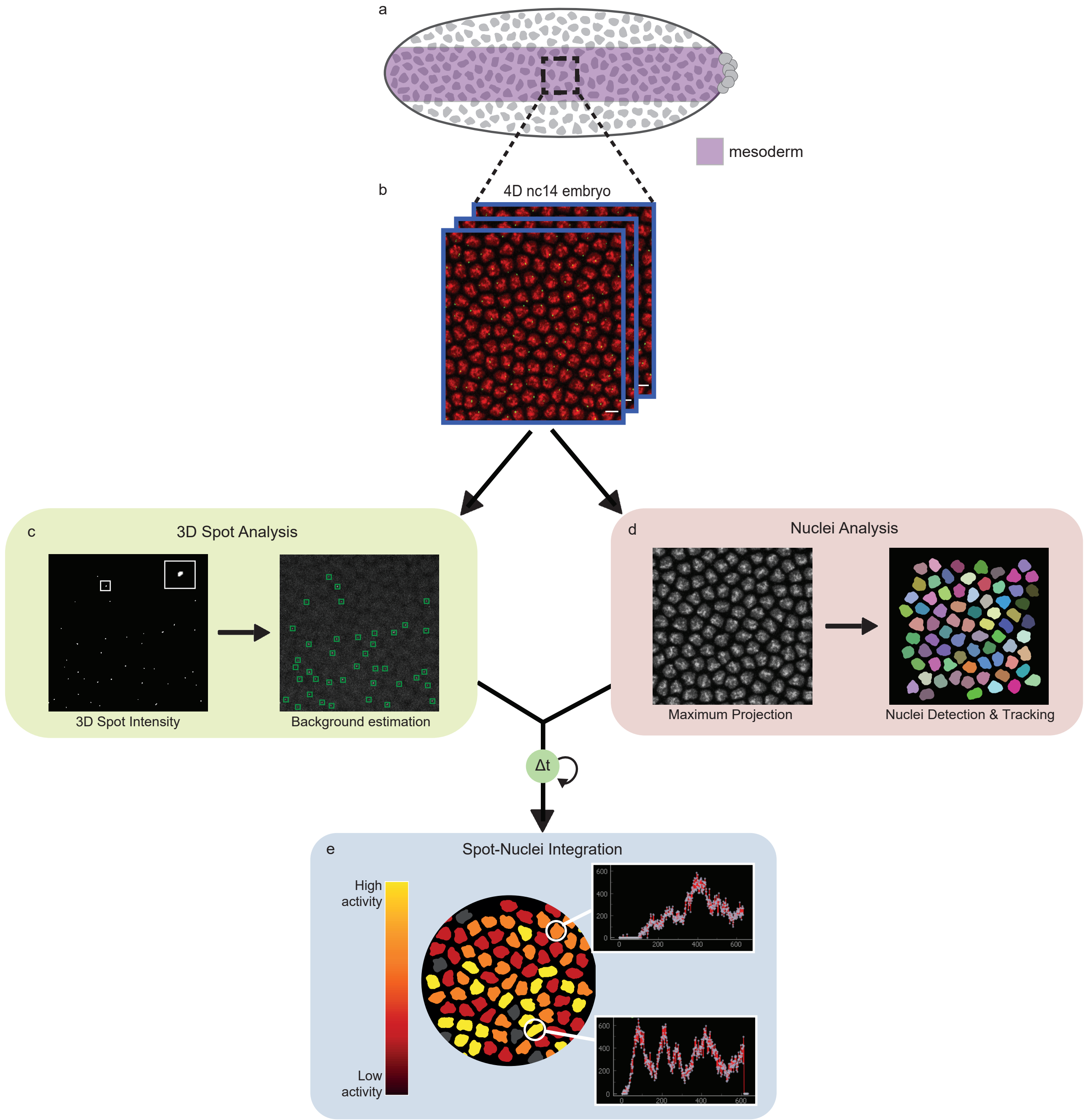

Supplementary Figure 2: Image analysis pipeline for retrieving single nuclei transcriptional activities

A) Schematic of the imaging set-up indicating spatial restriction to presumptive mesoderm (purple). B)Representation of Z-series showing nuclei (red) and MS2/MCP-GFP puncta (green). Scale bar is 5µm. C) Spot analysis is performed in 3D for each time point in the Z series. Inset shows zoom on single detected spot in software interface. Background estimation is performed independently for each spot using an average intensity value of the surrounding pixels (green rectangle). D) Nuclei analysis is performed in 2D for each time point. Nuclei are maximum projected, smoothed and detected, and then tracked between time points. Nuclei at the border are removed. E) Spot and nuclei information are integrated to associate each nuclei with its nearest transcription site for each time point in the Z-series, which can be mapped to each nuclei within the pattern. Inactive nuclei in the schematic are shown in grey, and highly active in yellow.

Supplementary Figure 3

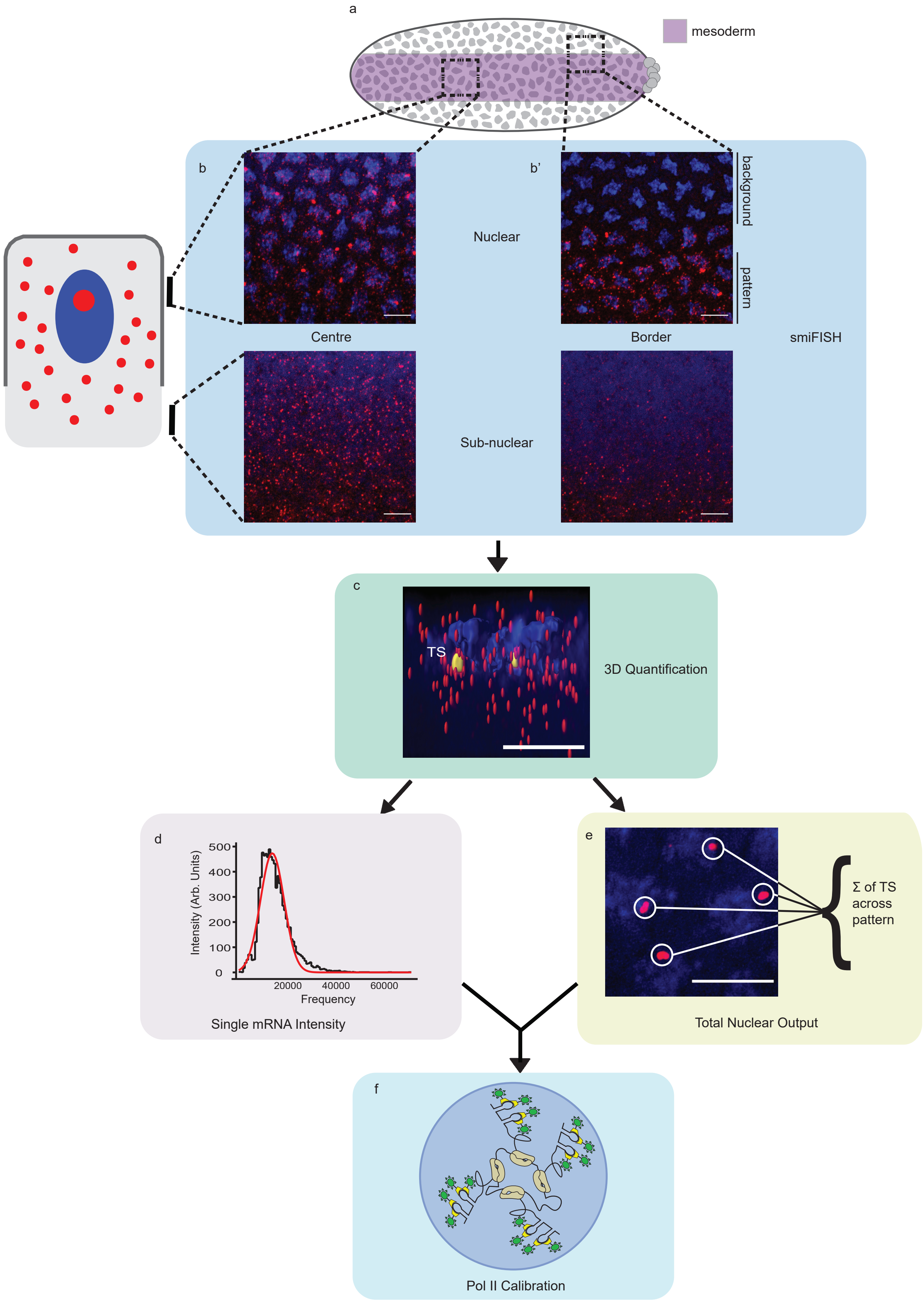

Supplementary Figure 3: Image analysis pipeline for single molecule FISH
A) Schematic of the embryo showing center and border imaging regions at the presumptive mesoderm (purple). B) Single molecule inexpensive fluorescence *in situ* hybridization (smiFISH; ref. 83) was used to identify individual mRNAs as well as the transcription site at the nuclei. Images are of a 1µm Z stack centred on the nuclear and subnuclear regions with DAPI in blue and mRNA in red. Scale bar is 5µm. C) 3D quantification of single molecule and transcription site intensity with Imaris (v9.2.2). Intensities of single molecules (red) and transcription sites (TS, yellow) around the nuclei (blue) were independently measured. Scale bar indicates 5 µm. D) Establishment of the median single molecule intensity for the entire Z-stack. E) Transcription site intensities across the pattern were summed and divided by the number of active nuclei such that sister chromatids were resolved as a single site, and the average TS intensity was established (ref. 85). F) The mean TS intensity of a Z-stack was divided by the median single molecule intensity of the Z stack to give an average number of transcripts per transcription site. At least 3 independent replicates per genotype were analyzed. Scale bar indicates 5 µm.

Supplementary Figure 4

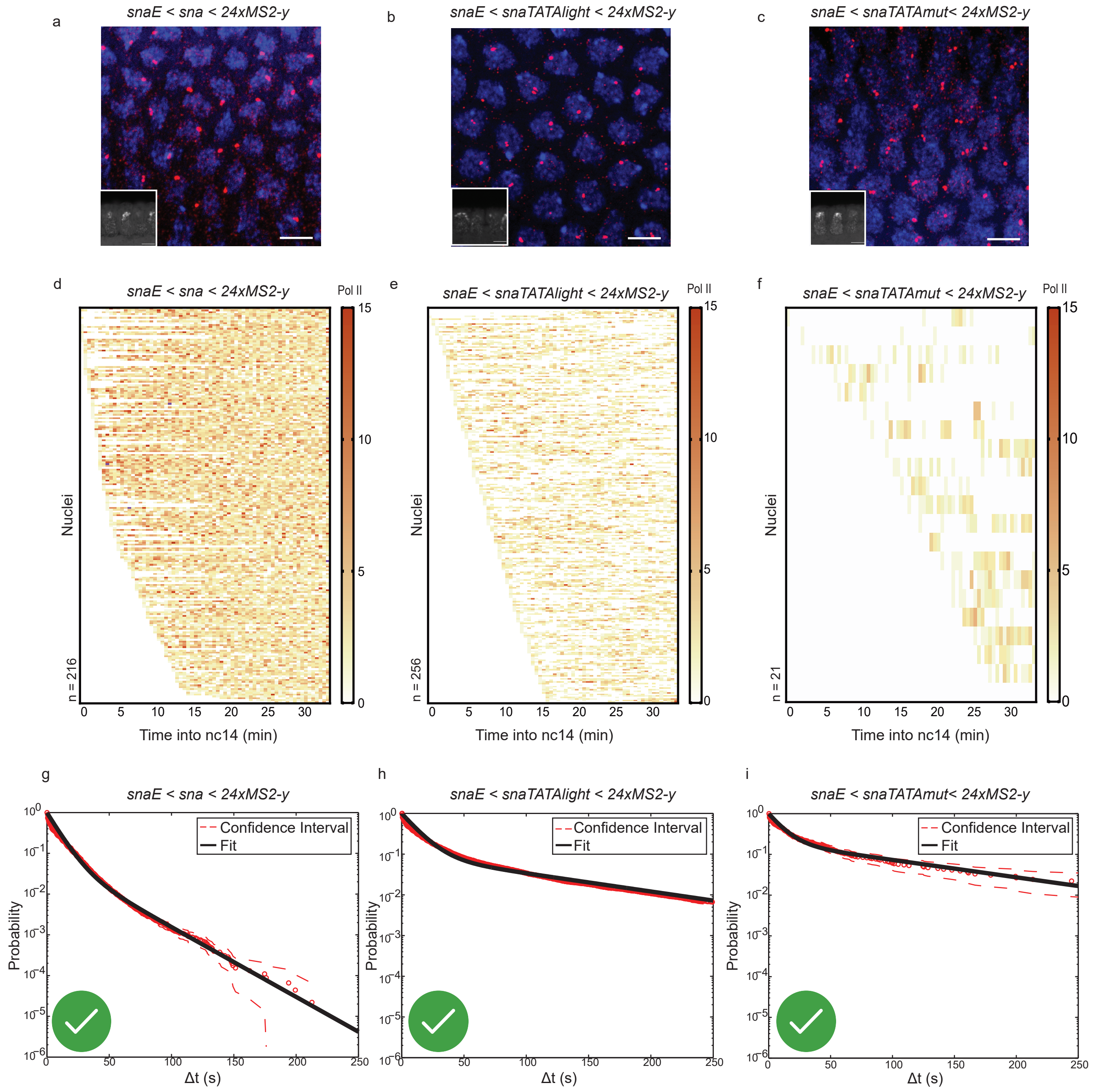

Supplementary Figure 4: Impact of the TATA box on sna promoter dynamics. A-C) Nuclear cycle 14 embryos, expressing one copy of the *sna* (A), *snaTATAlight* (B) or the *snaTATAmut* (C) transgene stained with yellow probes (smiFISH) and DAPI. Images are representative confocal 3 μm z-stack at the plane of the nuclei to show transcription site foci. Note that in some nuclei, two spots presumably emanating from two apposed sister chromatids are observable. Insets in A, B and C: zoomed view showing membrane invagination. Scale bar is 5 μm in all images. D-F) Heatmap showing frequency of Pol II initiation events per 30s window during the first 30 minutes of nc14 for each of the genotypes indicated. G-I) Survival function of the distribution of waiting times between polymerase initiation events (red circles) and the two-exponential fitting of the population (black line) for *sna, snaTATAlight* and *snaTATAmut* estimated using the Kaplan-Meyer method. Red dashes indicate the 95% confidence interval based on the Greenwood's formula (see Supplemental Table 1). Green check indicates accepted fitting.

Statistics: *snaE<sna*, n = 8 for smiFISH, 216 nuclei, 3 movies for live imaging; *snaE<snaTATAlight,* n = 3 for smiFISH, 256 nuclei, 6 movies for live imaging; *snaE<snaTATAmut*, n = 6 embryos for smiFISH, 21 nuclei, 3 movies for live imaging. See Supplementary Movies 1-3 and Supplementary Table 2.

Supplementary Figure 5

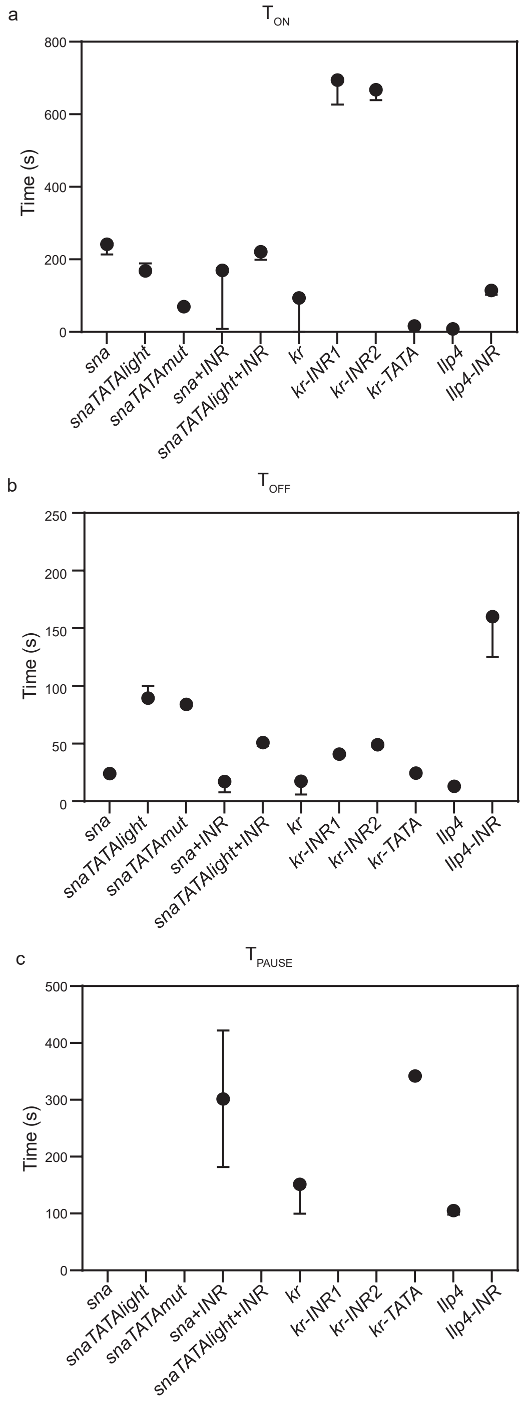

Supplementary Figure 5: Average State Durations and Probabilities
A) Average duration of the ON state of best fitting for indicated promoters. Error bars represent the smallest and largest values in optimal and close to optimal solutions (see Methods).
B) Average duration of the OFF state of best fitting for indicated promoters. Error bars represent the smallest and largest values in optimal and close to optimal solutions (see Methods).
C) Average duration of the PAUSE state of best fitting for indicated promoters. Error bars represent the smallest and largest values in optimal and close to optimal solutions (see Methods).

Supplementary Figure 6

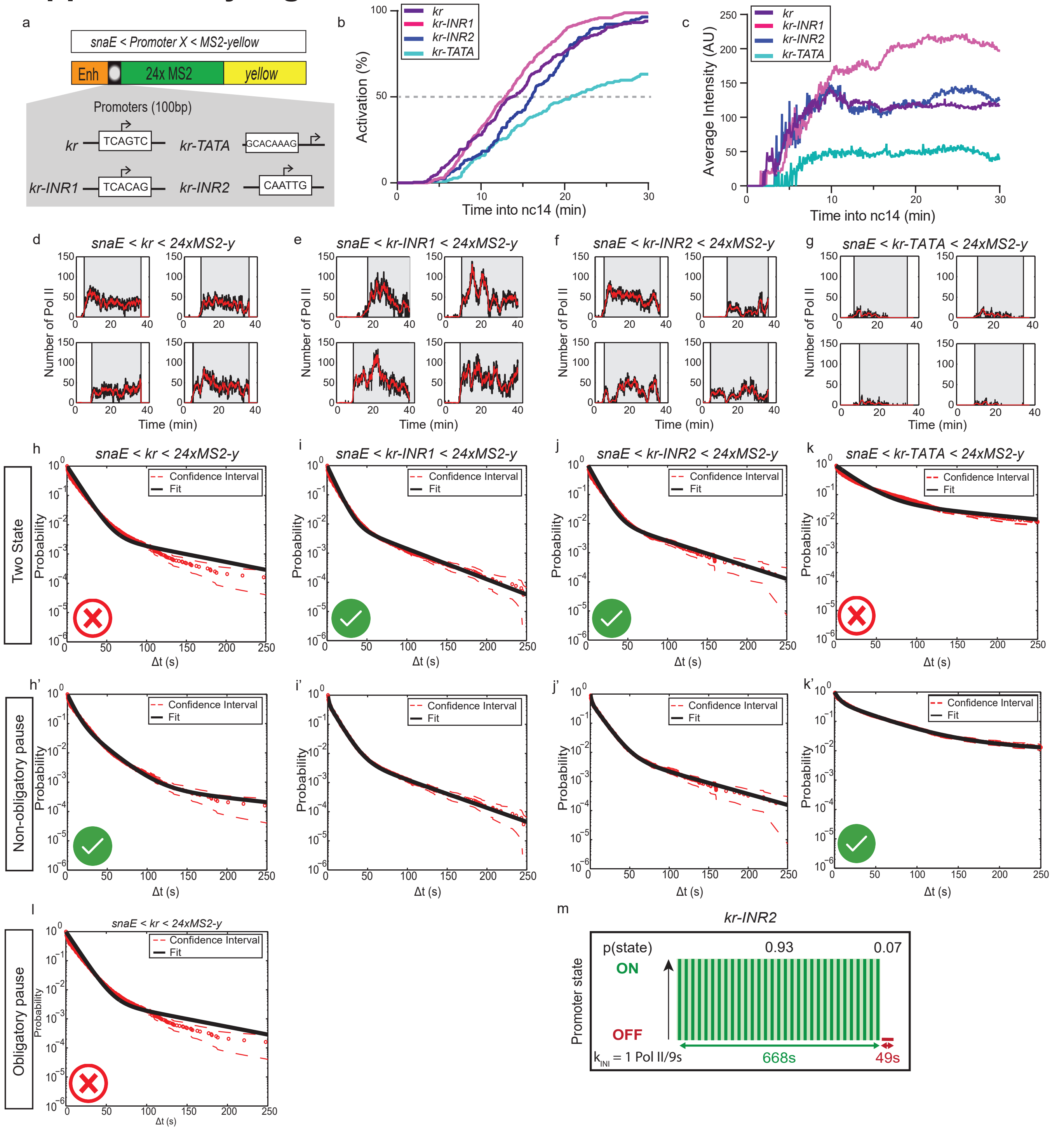

Supplementary Figure 6: Elucidating the impact of INR for kr promoter dynamics A) Schematic of the kr promoter transgenic variants. Two mutants of the INR motif were generated, *kr-INR1* corresponds to *kr* INR swap with the TSS region of *sna*, whereas *kr-INR2* is the swap with the TSS region of *brk*. *kr-TATA* contains the same TBP binding site mutation as the *snaTATAmut* promoter upstream of the indicated TSS. B) Cumulative activation curves of all nuclei during the first 30 minutes of nc14 for *kr* (purple), *kr-INR1* (pink), *kr-INR2* (blue) and *kr-TATA* (teal) embryos. Time zero is from anaphase during nc13-nc14 mitosis. C) Average instantaneous fluorescence of transcriptional foci of active nuclei during the first 30 minutes of nc14 for *kr* (purple), *kr-INR1* (pink), *kr-INR2* (blue) and *kr-TATA* (teal) embryos. Time zero is from anaphase during nc13-nc14 mitosis. D-G) Examples of individual activity events for the indicated genotypes. Red lines represent the reconstructed signal after Pol II positioning. H-K) Survival function of the distribution of waiting times between polymerase initiation events (red circles) and the two-exponential (H-K) and three exponential (H'-K') fitting of the population (black line) estimated using the Kaplan-Meyer method. Red dashes represent 95% confidence interval estimated based on the Greenwood's formula. (see also Supplementary Table 1). Green check indicates accepted fitting, red X indicates rejected fitting. L) Survival function of the distribution of waiting times between polymerase initiation events (red circles) and the three exponential obligatory pause model fitting of the population (black line) estimated using the Kaplan-Meyer method. Red dashes represent 95% confidence interval estimated based on the Greenwood's formula. M) Representation of the *kr-INR2* promoter dynamics. ON state durations are depicted in green and OFF states in red with state probabilities shown above.

Statistics: *snaE<kr*, 243 nuclei, 3 movies; *snaE<kr-INR1*, 342 nuclei, 4 movies; *snaE<kr-INR2*, 168 nuclei, 3 movies; *snaE<kr-TATA*, 98 nuclei, 2 movies. See Supplementary Movie 5-6 and Supplementary Table 2.

Supplementary Figure 7

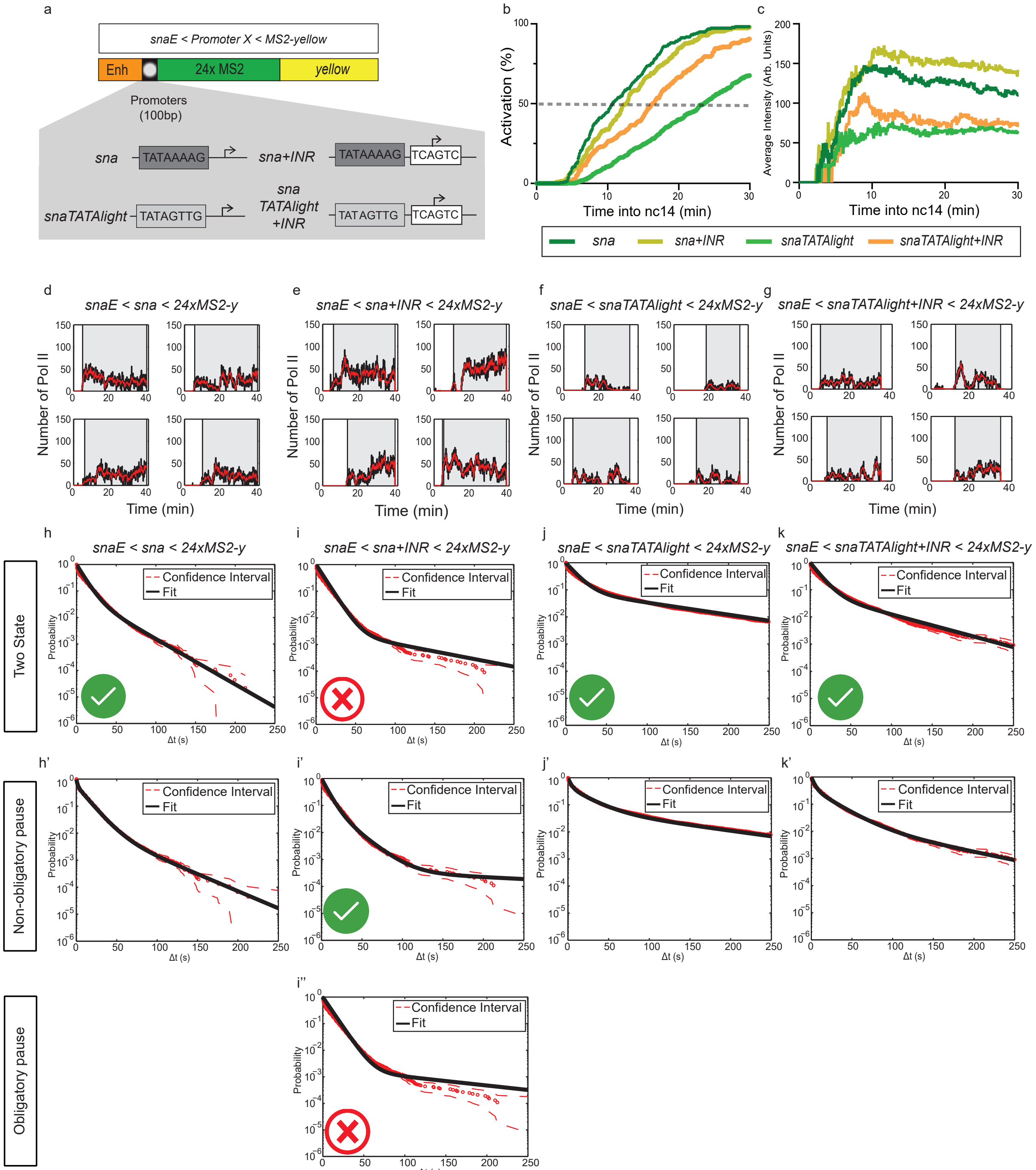

Supplementary Figure 7: Impact of the INR and TATA box on sna promoter dynamics. A) Scheme of the *sna* promoter and the 3 mutants generated to decode the role of the TATA box and the INR motif: *snaTATAlight, sna+INR, snaTATAlight+INR.* B)Cumulative activation curves of all nuclei during the first 30 minutes of nc14 for *sna* (green), *sna+INR* (yellow), *snaTATAlight* (light green) and *snaTATAlight+INR* (orange) embryos. Time zero is from anaphase during nc13-nc14 mitosis. C) Average instantaneous fluorescence of transcriptional foci of active nuclei during the first 30 minutes of nc14 for *sna* (green), *sna+INR* (yellow), *snaTATAlight* (light green) and *snaTATAlight+INR* (orange) embryos. Time zero is from anaphase during nc13-nc14 mitosis. D-G) Examples of single nuclei traces for the indicated genotypes. Red lines represent the signal after Pol II positioning. H-K) Survival function of the distribution of waiting times between polymerase initiation events (red circles) and the two-exponential (H-K), three exponential non-obligatory paused (H'-K') and three exponential obligatory pause (I") fitting of the population (black line) estimated using the Kaplan-Meyer method. Red dashes represent 95% confidence interval based on the Greenwood's formula (see also Supplementary Table 1). Green check indicates accepted fitting, red X indicates rejected fitting.

Statistics: *snaE<sna*, 216 nuclei, 3 movies; *snaE<sna+INR*, 236 nuclei, 4 movies; *snaE<snaTATAlight*, 353 nuclei, 6 movies; *snaE<snaTATAlight+INR*, 193 nuclei, 3 movies. See Supplementary Movies 1-2, 4 and Supplementary Table 2.

Supplementary Figure 8

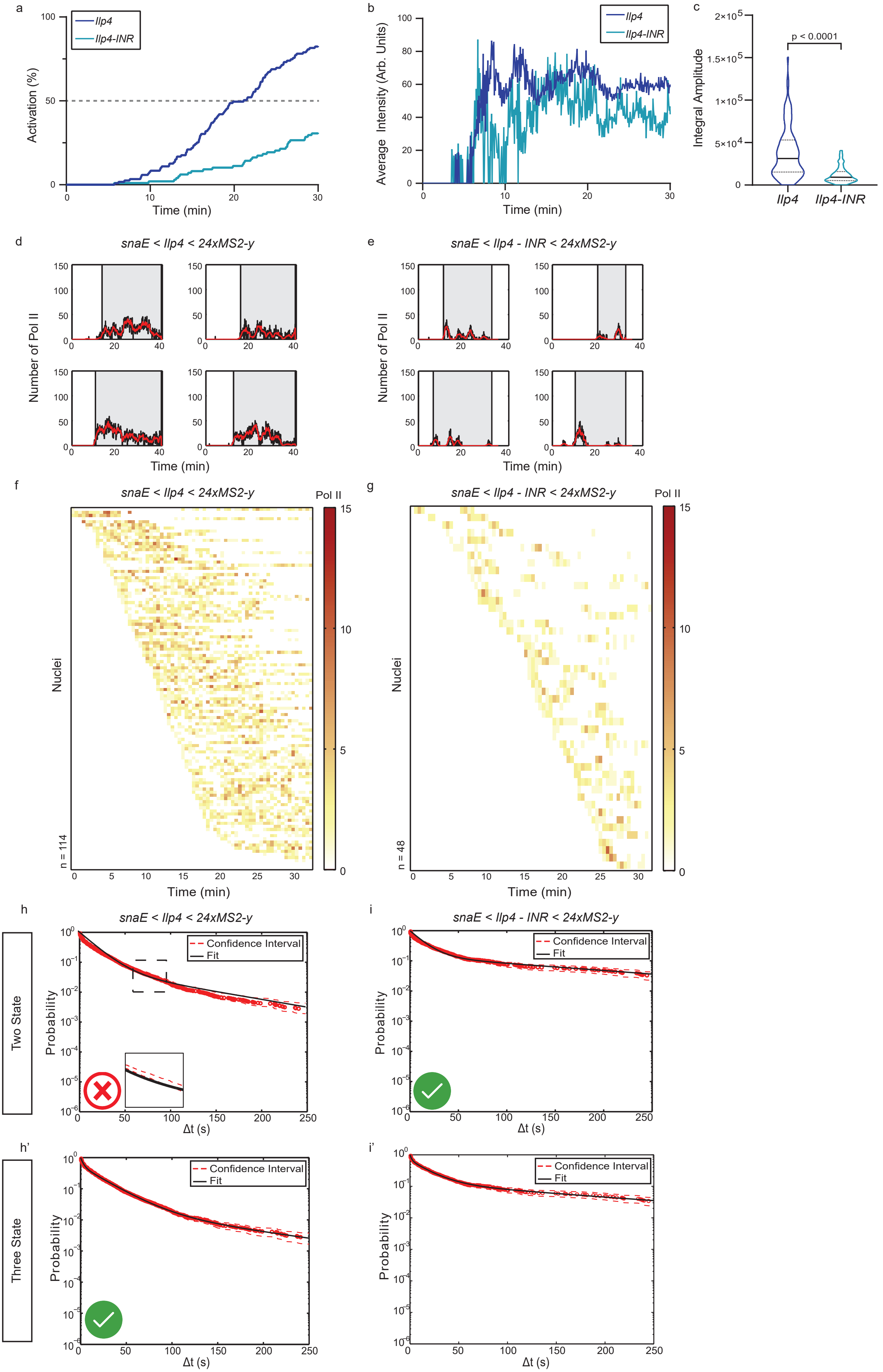

Supplementary Figure 8: Impact of the INR on Ilp4 promoter dynamics
A) Cumulative activation curves of all nuclei during the first 30 minutes of nc14 for *Ilp4* (dark blue) and *Ilp4-INR* (light blue) embryos. Time zero is from anaphase during nc13-nc14 mitosis. B) Average instantaneous fluorescence of transcriptional foci of active nuclei during the first 30 minutes of nc14 for *Ilp4* (dark blue) and *Ilp4-INR* (light blue) embryos. Time zero is from anaphase during nc13-nc14 mitosis. C) Distribution of individual trace integral amplitudes from first 30 minutes of nc14 for *Ilp4* (dark blue) and *Ilp4-INR* (light blue) embryos. Solid line represents median and dashed lines the first and third quartiles, using a one-sided Kruskal-Wallis test for significance with multiple comparison adjustment. D-E) Examples of single nuclei traces, for the indicated genotypes. Red lines represent the reconstructed signal after Pol II positioning.
F-G) Heatmaps indicating number of polymerases during nc14 for each genotype.
H-I) Survival function of the distribution of waiting times between polymerase initiation events (red circles) and the two-exponential (H-I) and three exponential (H'-I') fitting of the population (black line) estimated using the Kaplan-Meyer method. Red dashes represent 95% confidence interval based on the Greenwood's formula (see also Supplementary Table 1). Green check indicates accepted fitting, red X indicates rejected fitting.

Statistics: *snaE<Ilp4*, 114 nuclei, 2 movies; *snaE<Ilp4-INR*, 48 nuclei, 2 movies. See Supplementary Table 2.

Supplementary Figure 9

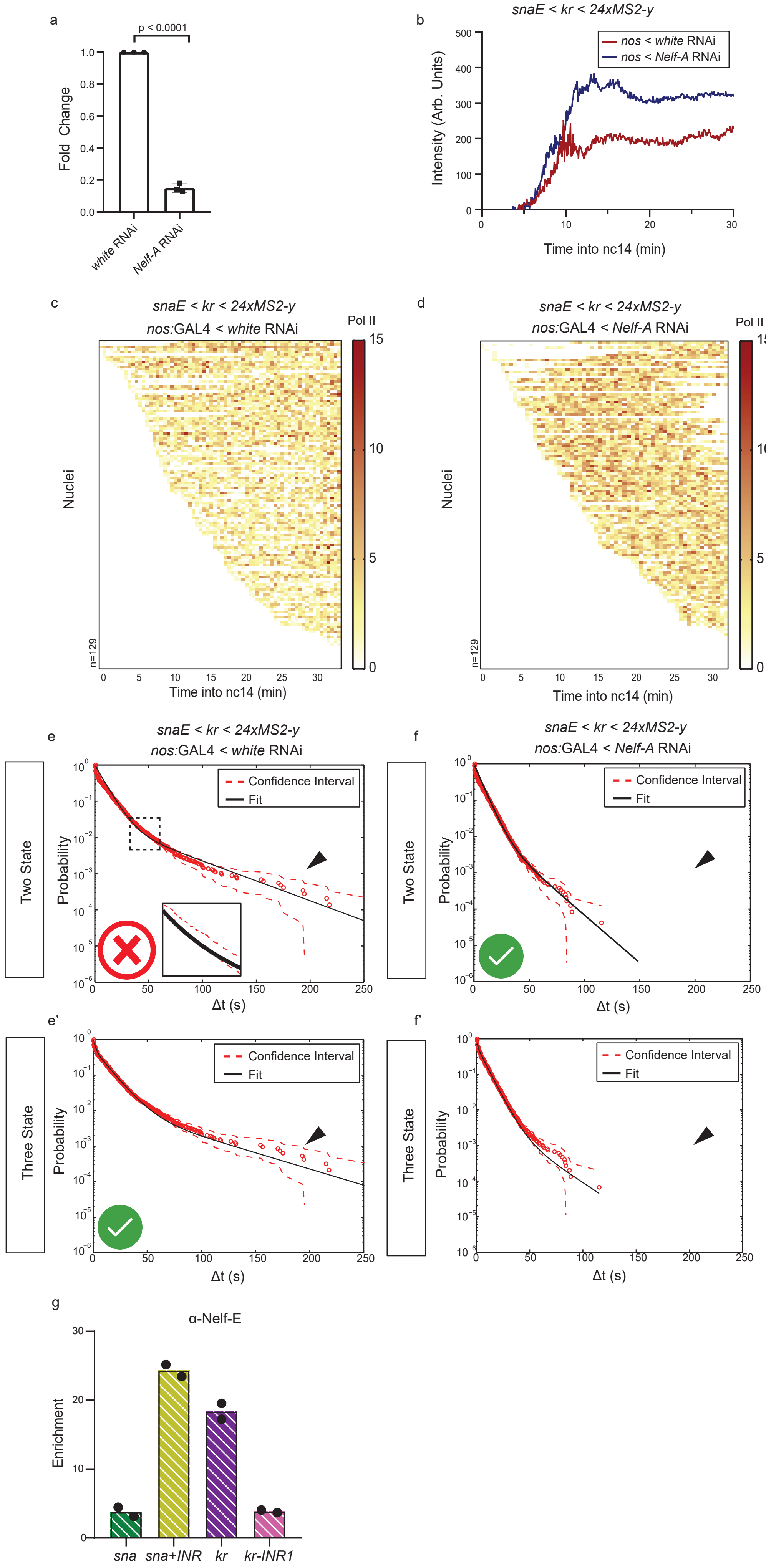

Supplementary Figure 9: Altering pausing in trans changes state dynamics
A) RNAi-mediated knockdown of Nelf-A transcript level measured by qRT-PCR (average ± SD) using a one-tailed Student's t-test, n=3. B) Average instantaneous fluorescence of transcriptional foci of active nuclei during the first 30 minutes of nc14 for *nos> white RNAi; kr* (red) and *nos>Nelf-A RNAi; kr* (blue) embryos. Time zero is from anaphase during nc13-nc14 mitosis. C-D) Heatmaps indicating number of polymerases during nc14 for each genotype. E-F) Survival function of the distribution of waiting times between polymerase initiation events (red circles) and the two-exponential (E-F) and three exponential (E'-F') fitting of the population (black line) estimated using the Kaplan-Meyer method. Red dashes represent 95% confidence interval estimated based on the Greenwood's formula (see also Supplementary Table 1). Green X indicates accepted fitting, red X indicates rejected fitting. Arrowheads indicate long duration waiting times. G) Enrichment of Nelf-E at promoter region of indicated transgenes using ChIP-qPCR, shown as average of n=3 biological replicates with individual replicates indicated.

Statistics: *nos:GAL4 < white RNAi; snaE<kr,* 129 nuclei, 2 movies;
nos:GAL4 < Nelf-A RNAi; snaE<kr, 129 nuclei, 2 movies. See Supplementary Movies 7-8.

Supplementary Figure 10

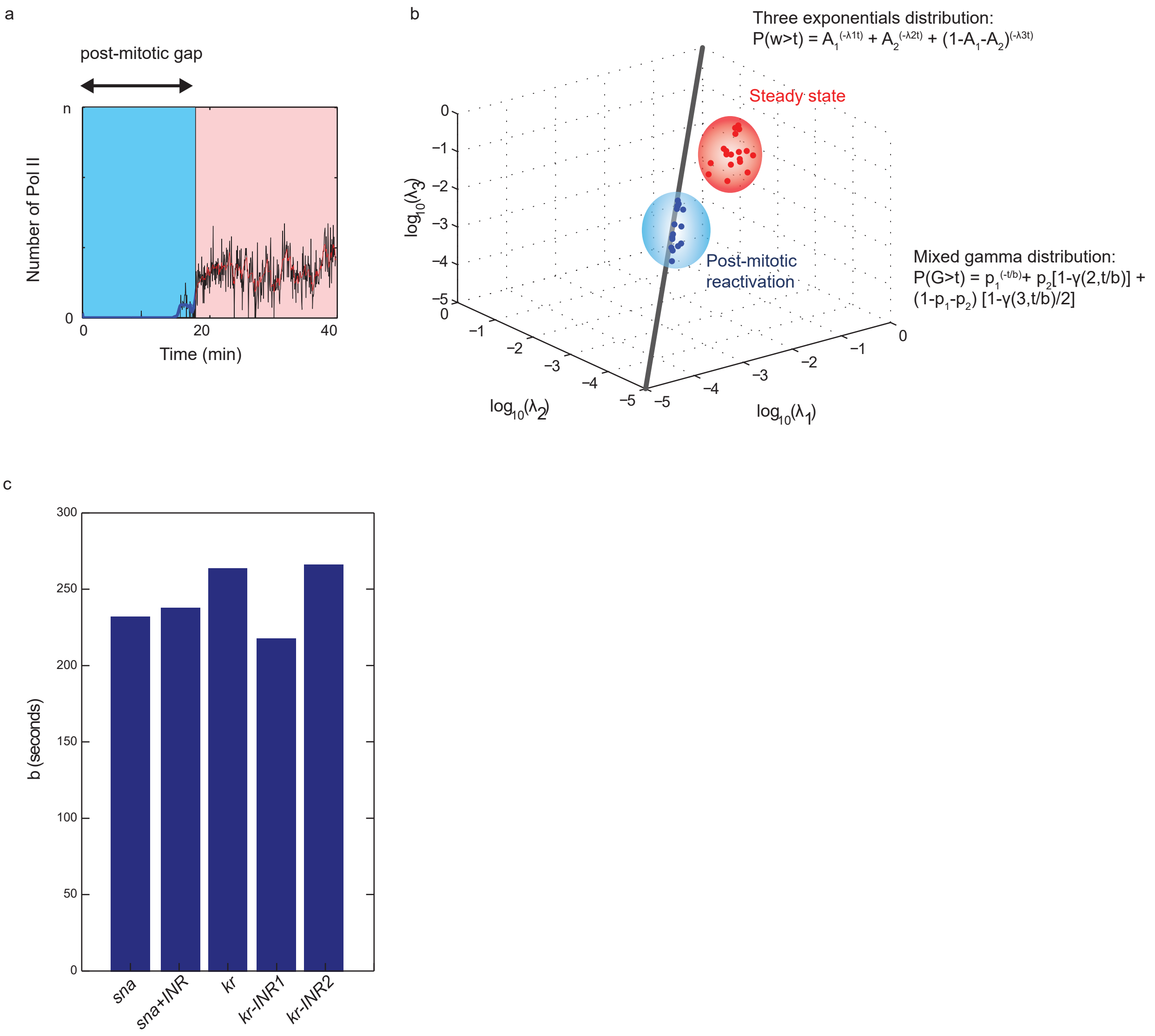

Supplementary Figure 10: Distribution of the post-mitotic gaps
A) Example trace indicating timeframe of post-mitotic gap (G, blue), i.e. the lag time between nc13 to nc14 mitosis to transcriptional activation in nc14. In comparison, the time window used to decipher bursting kinetics with the deconvolution method is shown in red. B) A three-exponential fit of the data from various promoters shows that the post-mitotic gaps (blue) have different timescales (inverses of λi) than the bursting (red) waiting time. The timescales of the post-mitotic gap duration are even ($\lambda_1 = \lambda_2 = \lambda_3 = 1/b$). Thus, a mixed gamma distribution fits well the distribution of the post-mitotic gap.
C) The values of the gap step durations (b) for different promoters.

Genotype	Two States Model		Three States Model	
	Objective Function	Kolmogorov-Smirnov p-value	Objective Function	Kolmogorov-Smirnov p-value
snaE>***sna*** >24xMS2-y	1.52E-02	7.17E-01	1.37E-03	9.25E-01
snaE>***snaTATAlight*** >24xMS2-y	1.01E-02	5.99E-04	1.35E-03	3.70E-01
snaE>***snaTATAmut*** >24xMS2-y	2.50E-02	8.20E-01	1.63E-03	9.58E-01
snaE>***sna+INR*** >24xMS2-y	4.81E-02	1.37E-04	1.17E-02	9.99E-01
snaE>***snaTATAlight+INR*** >24xMS2-y	1.94E-02	5.99E-04	2.16E-03	9.97E-01
snaE>***kr*** >24xMS2-y	4.48E-02	2.15E-06	8.58E-03	5.08E-01
snaE>***kr-INR1*** >24xMS2-y	1.73E-02	9.41E-01	1.58E-03	1.00E+00
snaE>***kr-INR2*** >24xMS2-y	2.06E-02	9.80E-01	1.95E-03	1.00E+00
snaE>***kr-TATA*** >24xMS2-y	2.45E-02	4.21E-16	7.08E-04	9.78E-01
snaE>***Ilp4*** >24xMS2-y	1.86E-02	1.42E-08	8.39E-04	9.98E-01
snaE>***Ilp4-INR*** >24xMS2-y	7.40E-03	9.02E-01	1.75E-03	1.00E+00
nos :GAL4; RNAi *white,* snaE>***kr*** >24xMS2-y	1.99E-02	5.26E-14	1.92E-03	2.37E-01
nos :GAL4; RNAi *Nelf-A* , snaE>***kr*** >24xMS2-y	1.52E-02	2.52E-02	2.95E-03	6.74E-01

Supplementary Table 1: Objective functions and one-sided Kolmogorov-Smirnov test results for promoters derived from deconvolution and multi-exponential regression fitting of live imaging data. Parameters are provided for the most parsimonious fitting of each genotype. Bold indicates the most parsimonious appropriate fitting of the data. A two-state model was considered rejected when the p-value of the Kolmogorov-Smirnov test was less than $5.00e^{-4}$. *As the confidence interval criterion rejected and the KS criterion accepted the two-state model for the *SnaTATAlight+INR* promoter, this genotype was considered borderline.

Genotype	Two States Model Parameters							
	**k1+**	**k1-**	**k2**	**T(Off) (s)**	**T(On) (s)**	**Pol II Initiation (s/event)**	**p(OFF)**	**p(ON)**
snaE>***sna*** >24xMS2-y	**4.17E-02**	**4.14E-03**	**1.13E-01**	**24**	**242**	**8.87**	**0.09**	**0.91**
Minimum	4.17E-02	4.14E-03	1.13E-01	24	242	8.87	0.09	0.90
Maximum	4.31E-02	4.67E-03	1.16E-01	23	214	8.61	0.10	0.91
snaE>***snaTATAlight*** >24xMS2-y	**1.12E-02**	**5.94E-03**	**7.64E-02**	**89**	**168**	**13.09**	**0.35**	**0.65**
Minimum	9.98E-03	5.29E-03	7.29E-02	100	189	13.72	0.35	0.65
Maximum	1.12E-02	5.94E-03	7.64E-02	89	168	13.09	0.35	0.65
snaE>***snaTATAmut*** >24xMS2-y	**1.19E-02**	**1.43E-02**	**7.74E-02**	**84**	**70**	**12.91**	**0.55**	**0.45**
Minimum	1.19E-02	1.43E-02	7.74E-02	84	70	12.91	0.55	0.45
Maximum	1.19E-02	1.43E-02	7.75E-02	84	70	12.91	0.55	0.45
snaE>***snaTATAlight+INR*** >24xMS2-y	**1.97E-02**	**4.53E-03**	**8.62E-02**	**51**	**221**	**11.61**	**0.19**	**0.81**
Minimum	1.97E-02	4.53E-03	8.62E-02	51	221	11.61	0.19	0.81
Maximum	2.09E-02	5.03E-03	8.95E-02	48	199	11.17	0.19	0.81
snaE>***kr-INR1*** >24xMS2-y	**2.44E-02**	**1.44E-03**	**1.34E-01**	**41**	**694**	**7.46**	**0.06**	**0.94**
Minimum	2.44E-02	1.44E-03	1.34E-01	41	694	7.46	0.06	0.94
Maximum	2.52E-02	1.60E-03	1.38E-01	40	627	7.26	0.06	0.94
snaE>***kr-INR2*** >24xMS2-y	**2.04E-02**	**1.50E-03**	**1.14E-01**	**49**	**668**	**8.75**	**0.07**	**0.93**
Minimum	2.04E-02	1.50E-03	1.14E-01	49	668	8.75	0.07	0.93
Maximum	2.10E-02	1.56E-03	1.16E-01	48	639	8.62	0.07	0.93
snaE>***Ilp4-INR*** >24xMS2-y	**6.27E-03**	**8.81E-03**	**6.37E-02**	**160**	**114**	**15.70**	**0.58**	**0.42**
Minimum	6.27E-03	8.81E-03	6.37E-02	160	114	15.70	0.55	0.42
Maximum	7.99E-03	9.84E-03	6.74E-02	125	102	14.84	0.58	0.45

Genotype	Three States Model Parameters											
	**k1+**	**k1-**	**k2+**	**k2-**	**k3**	**T(OFF) (s)**	**T(PAUSE) (s)**	**T(On) (s)**	**Pol II Initiation (s/event)**	**p(OFF)**	**p(PAUSE)**	**p(ON)**
snaE>***sna+INR*** >24xMS2-y	**3.35E-03**	**4.61E-04**	**5.75E-02**	**5.92E-03**	**1.31E-01**	**17**	**302**	**171**	**7.63**	**0.09**	**0.01**	**0.90**
Minimum	2.38E-03	2.71E-04	5.75E-02	5.89E-03	1.31E-01	17	422	171	7.63	0.09	0.01	0.51
Maximum	5.52E-03	4.61E-04	1.26E-01	1.17E-01	2.77E-01	8	182	9	3.61	0.47	0.03	0.90
snaE>***kr*** >24xMS2-y	**6.61E-03**	**1.25E-04**	**5.77E-02**	**1.05E-02**	**1.32E-01**	**17**	**151**	**95**	**7.58**	**0.15**	**0.02**	**0.83**
Minimum	6.60E-03	1.25E-04	5.76E-02	1.05E-02	1.32E-01	17	151	95	7.58	0.15	0.02	0.00
Maximum	1.00E-02	1.62E-01	1.66E-01	7.16E+01	6.00E+01	6	100	0	0.02	0.96	0.04	0.83
snaE>***kr-TATA*** >24xMS2-y	**2.92E-03**	**2.45E-03**	**4.08E-02**	**5.81E-02**	**1.11E-01**	**25**	**342**	**17**	**9.02**	**0.44**	**0.26**	**0.31**
Minimum	2.92E-03	2.45E-03	4.08E-02	5.81E-02	1.11E-01	25	342	17	9.02	0.44	0.26	0.31
Maximum	2.92E-03	2.45E-03	4.08E-02	5.81E-02	1.11E-01	25	342	17	9.02	0.44	0.26	0.31
snaE>***Ilp4*** >24xMS2-y	**9.53E-03**	**3.44E-03**	**7.59E-02**	**1.17E-01**	**1.75E-01**	**13**	**105**	**8**	**5.71**	**0.53**	**0.12**	**0.34**
Minimum	9.53E-03	3.29E-03	7.49E-02	1.11E-01	1.73E-01	13	105	9	5.79	0.53	0.11	0.34
Maximum	1.02E-02	3.44E-03	7.60E-02	1.17E-01	1.75E-01	13	98	8	5.71	0.53	0.12	0.36

Supplementary Table 2: Kinetic parameters for promoters derived from deconvolution and multi-exponential regression fitting of live imaging data. Parameters are provided for the most parsimonious fitting of each genotype, with the optimal fitting highlighted in grey. Minimum and maximum values indicate the boundaries of the error interval. State durations are calculated from the provided switching rates (k_n) and time durations for each state are provided as 'T(*state*)'. State probability values are indicated as 'p(*state*)'. All calculations are provided in Methods.

Promoter	Sequence
sna	GACAGCGGCGTCGGCAGAGGCGCAGAGTTCCGGGTATAAAAGAGCGTGCTCGACTGTTGACCTGTCACAGCCACCTCAGCTCTCGTTGAGAACGCAACCA
snaTATAlight	GACAGCGGCGTCGGCAGAGGCGCAGAGTTCCGGGTATAGTTGAGCGTGCTCGACTGTTGACCTGTCACAGCCACCTCAGCTCTCGTTGAGAACGCAACCA
snaTATAmut	GACAGCGGCGTCGGCAGAGGCGCAGAGTTCCGGGGCACAAAGAGCGTGCTCGACTGTTGACCTGTCACAGCCACCTCAGCTCTCGTTGAGAACGCAACCA
sna+INR	GACAGCGGCGTCGGCAGAGGCGCAGAGTTCCGGGTATAAAAGAGCGTGCTCGACTGTTGACCTGTCAGTCCCACCTCAGCTCTCGTTGAGAACGCAACCA
snaTATAlight+INR	GACAGCGGCGTCGGCAGAGGCGCAGAGTTCCGGGTATAGTTGAGCGTGCTCGACTGTTGACCTGTCAGTCCCACCTCAGCTCTCGTTGAGAACGCAACCA
kr	TTCGCCGAGACAGAGCGTACTTATAGTTAGCTCACGCAGCGAATTTGTATCAGTCGTGATTTGGCTCTGTCAGCGAAAGGAACAACCATTTGTTGTGCC
kr-INR1	TTCGCCGAGACAGAGCGTACTTATAGTTAGCTCACGCAGCGAATTTGTATCACAGGTGATTTGGCTCTGTCAGCGAAAGGAACAACCATTTGTTGTGCC
kr-INR2	TTCGCCGAGACAGAGCGTACTTATAGTTAGCTCACGCAGCGAATTTGTACAATTGGTGATTTGGCTCTGTCAGCGAAAGGAACAACCATTTGTTGTGCC
kr-TATA	TTCGCCGAGACAGAGCGTACTGCACAAAGGCTCACGCAGCGAATTTGTATCAGTCGTGATTTGGCTCTGTCAGCGAAAGGAACAACCATTTGTTGTGCC
brk	AGGCGAGGCAGTCTAGAACGTATCTTCGAACTAGAACGCCGAGGGCAGAACAATTGTGTTTTGGATTCCTTGCCGTGCGGATCGCAAACGCGAGTTATGA
Ilp4	AAAGTCAATTAGCGAGTCAACATTTTGAGCGCCGGCCAACTCCAAGGATCAGTATCATTTGGCATGCCCAGCGATCGGTTTGCCAAGAGCACGAGAAGTT
Ilp4-INR	AAAGTCAATTAGCGAGTCAACATTTTGAGCGCCGGCCAACTCCAAGGATCACAGTCATTTGGCATGCCCAGCGATCGGTTTGCCAAGAGCACGAGAAGTT

Supplementary Table 3: Promoter sequences for the genotypes listed in this study.

Probe	Sequence
Probe 1	TTA ACC TTG ATG CTG ATG ATG CCA CCA CTT ACA CTC GGA CCT CGT CGA CAT GCA TT
Probe 2	GTG GGA AAT AGA TGG GGA CCA CTT GTC TCG TTA CAC TCG GAC CTC GTC GAC ATG CAT T
Probe 3	ATG CCA TGA AAT TGC GGT GAG TAC GGC ATT TAC ACT CGG ACC TCG TCG ACA TGC ATT
Probe 4	ATG AGT GCC AGC AAC CCA CTG CAT TTT TAC ACT CGG ACC TCG TCG ACA TGC ATT
Probe 5	ATC CTC GTG GAT ACG GCA AAT TGT CGA TGA TTA CAC TCG GAC CTC GTC GAC ATG CAT T
Probe 6	GAC ATT TAC CGC ATA GGG GCA CGG ATT AGT TAC ACT CGG ACC TCG TCG ACA TGC ATT
Probe 7	CCA ATC TGG ATA CGG AAT TAG CTC CGG TGT TAC ACT CGG ACC TCG TCG ACA TGC ATT
Probe 8	GTG TTC GAC TCC AAC AGG TAG AGC ATT TTG CTT ACA CTC GGA CCT CGT CGA CAT GCA TT
Probe 9	GAT CAG GGT CAC AAG GAT CCA CCC TTT GTT ACA CTC GGA CCT CGT CGA CAT GCA TT
Probe 10	TTT CTG TGG CAA GAC AGG ACG ATA TTG TTT CGT TAC ACT CGG ACC TCG TCG ACA TGC ATT
Probe 11	GTT TTG GTA TTG AAA CGG TAT TTG GCG GCC CAT TAC ACT CGG ACC TCG TCG ACA TGC ATT
Probe 12	CTC ATC ACA CGT GAA GTG GTA TGG GAG TTT GGT TAC ACT CGG ACC TCG TCG ACA TGC ATT
Probe 13	TCT CCA GGA CTT GTT CAG TTC CCA GGA GTT ACA CTC GGA CCT CGT CGA CAT GCA TT
Probe 14	GAT TTG TGT CCA CGC CAG GTA GCT CGT ATT ACA CTC GGA CCT CGT CGA CAT GCA TT
Probe 15	TCC GAA TTC GCG TAT CCG TGG TCA AGT CAT TAC ACT CGG ACC TCG TCG ACA TGC ATT
Probe 16	GTG GTA TTG CCG ATG CCC ACG GTT CCT TAC ACT CGG ACC TCG TCG ACA TGC ATT
Probe 17	GTT GGC GCA ATC TCC AGC TGT ATT TGA GTT ACA CTC GGA CCT CGT CGA CAT GCA TT
Probe 18	GGC TCC AAC TAT ATC GCT CCT GAA GTT TTT ACA CTC GGA CCT CGT CGA CAT GCA TT
Probe 19	CCC GTC AAA CTG CGG TCC ATG TTT ATA TAG TTA CAC TCG GAC CTC GTC GAC ATG CAT T
Probe 20	CCT CCA CTC CTG CAG GAC CCA CAG AAT TAC ACT CGG ACC TCG TCG ACA TGC ATT
Probe 21	AGA TTG GCG GGC ATT CAC ATA AGT TTT TAA CCT TAC ACT CGG ACC TCG TCG ACA TGC ATT
Probe 22	TGT AGA GAC ACT AAT ACT GGA GAC TAC ATT GCT TAC ACT CGG ACC TCG TCG ACA TGC ATT
Probe 23	GAA TTG GCG GGC AAA TAA GTG CGA CTT GGA GTT ACA CTC GGA CCT CGT CGA CAT GCA TT
Probe 24	GGC GTT ATT CCT CAA ATC ACA CAC AGT ATT CTT ACA CTC GGA CCT CGT CGA CAT GCA TT

Supplementary Table 4: smiFISH probe sequences for single molecule detection of the *yellow* mRNA sequence.

Primer	Sequence
UniversalSnaEnFwd	CCATGTGTTGCTGGGAAATC
KrPrRev	ACCGTAGGATCCGGCACAACA
SnaPrRev	GTACCGTAGGATCCTGGTTGCGT
Ilp4PrRev	GTACCGTAGGATCAACTTCTCG
GeneDesertFwd	AATTGCATCGCAACACAATGAG
GeneDesertRev	TCGTGAAATGTTTGCTACTGGAAT
YellowFwd	GAAAACAGACAGCGATAACTTGC
YellowRev	GGAGAATTAGCAGGGCCAAAC
NelfAFwd[84]	ATGGCGAACGTAAGGGACAG
NelfARev[84]	GGAGCCGTTTATCCACGAGTC
qPCRPosCtrlFwd[57]	ACCCAATGCCACAATTAGCG
qPCRPosCtrlRev[57]	AACTGTTGCTGCGTCGATTG

Supplementary Table 5: Primer sequences used in this study.
