# Supplementary material for: Quantitative imaging of transcription in living Drosophila embryos reveals the impact of core promoter motifs on promoter state dynamics
Source: Nat Commun. 2021 Jul 23;12:4504. doi: 10.1038/s41467-021-24461-6 (PMC8302612; doi:10.1038/s41467-021-24461-6)
Supplement: Supplementary file 3 — Description of Additional Supplementary Files [file 41467_2021_24461_MOESM3_ESM.pdf]

Description of Additional Supplementary Files

File Name: Supplementary Movie 1
Description: Representative false-colour imaging of snaE<sna<24xMS2-yellow in nc14. Nuclei colour corresponds to GFP fluorescence intensity as in Figure 3C with inactive nuclei

File Name: Supplementary Movie 2
Description: Representative false colour imaging of snaE<snaTATAlight<24xMS2- yellow in nc14. Nuclei colour corresponds to GFP fluorescence intensity as in Figure 3C with inactive nuclei in grey and highly active nuclei in yellow. Intensity scale is common to Supplementary Movies 1-3.

File Name: Supplementary Movie 3
Description: Representative false colour imaging of snaE<snaTATAmut<24xMS2- yellow in nc14. Nuclei colour corresponds to GFP fluorescence intensity as in Figure 3C with inactive nuclei in grey and highly active nuclei in yellow. Intensity scale is common to Supplementary Movies 1-3.

File Name: Supplementary Movie 4
Description: Live imaging of snaE<sna+INR<24xMS2-yellow representative of NC14 beginning at mitosis. Nuclei are detected using His2Av-mRFP and MS2 using MCP-GFP.

File Name: Supplementary Movie 5
Description: Live imaging of snaE<kr<24xMS2-yellow representative of NC14 beginning at mitosis. Nuclei are detected using His2Av-mRFP and MS2 using MCP-GFP.

File Name: Supplementary Movie 6
Description: Live imaging of snaE<kr-INR1<24xMS2-yellow representative of NC14 beginning at mitosis. Nuclei are detected using His2Av-mRFP and MS2 using MCP-GFP.

File Name: Supplementary Movie 7
Description: Live imaging of snaE<kr<24xMS2-yellow in a nanos:GAL4> UAS:MCP- GFP-His2Av-RFP, UAS:white RNAi background, representative of nc14 beginning at mitosis. Nuclei are detected using His2Av-mRFP and MS2 using MCP-GFP.

File Name: Supplementary Movie 8
Description: Live imaging of snaE<kr<24xMS2-yellow in a nanos:GAL4> UAS:MCP- GFP-His2Av-RFP, UAS:Nelf-A RNAi background, representative of nc14 beginning at mitosis. Nuclei are detected using His2Av-mRFP and MS2 using MCP-GFP.
