# Supplementary material for: Quantitative imaging of transcription in living Drosophila embryos reveals the impact of core promoter motifs on promoter state dynamics
Source: Nat Commun. 2021 Jul 23;12:4504. doi: 10.1038/s41467-021-24461-6 (PMC8302612; doi:10.1038/s41467-021-24461-6)
Supplement: Supplementary file 12 — Reporting Summary [file 41467_2021_24461_MOESM12_ESM.pdf]

nature research

Corresponding author(s): LAGHA, Mounia

Last updated by author(s): Feb 26, 2021

Reporting Summary

Nature Research wishes to improve the reproducibility of the work that we publish. This form provides structure for consistency and transparency in reporting. For further information on Nature Research policies, see our Editorial Policies and the Editorial Policy Checklist.

Statistics

For all statistical analyses, confirm that the following items are present in the figure legend, table legend, main text, or Methods section.

n/a	Confirmed	
☐	☒	The exact sample size (*n*) for each experimental group/condition, given as a discrete number and unit of measurement
☐	☒	A statement on whether measurements were taken from distinct samples or whether the same sample was measured repeatedly
☐	☒	The statistical test(s) used AND whether they are one- or two-sided<br>*Only common tests should be described solely by name; describe more complex techniques in the Methods section.*
☐	☒	A description of all covariates tested
☐	☒	A description of any assumptions or corrections, such as tests of normality and adjustment for multiple comparisons
☐	☒	A full description of the statistical parameters including central tendency (e.g. means) or other basic estimates (e.g. regression coefficient) AND variation (e.g. standard deviation) or associated estimates of uncertainty (e.g. confidence intervals)
☐	☒	For null hypothesis testing, the test statistic (e.g. *F*, *t*, *r*) with confidence intervals, effect sizes, degrees of freedom and *P* value noted<br>*Give P values as exact values whenever suitable.*
☒	☐	For Bayesian analysis, information on the choice of priors and Markov chain Monte Carlo settings
☒	☐	For hierarchical and complex designs, identification of the appropriate level for tests and full reporting of outcomes
☒	☐	Estimates of effect sizes (e.g. Cohen's *d*, Pearson's *r*), indicating how they were calculated

Our web collection on statistics for biologists contains articles on many of the points above.

Software and code

Policy information about availability of computer code

Data collection	Live and fixed sample data were collected using 3D Zen Black (v2.3) for Airyscan processing (Zeiss).
Data analysis	Fixed sample analysis was performed using Imaris v9.2.2 (Oxford Instruments).<br><br>Extraction and analysis of live imaging data was initially performed with a custom Python (v3.6) software package detailed in the Materials and Methods. Software code and an overview of its function is available at http://www.igmm.cnrs.fr/segment-track/.<br><br>Mathematical modelling of burst parameters and multi-exponential regression fitting was performed with custom Matlab (R2020a) software using equations described in the Methods section. Software code is available and will be published in a separate manuscript (Tantale et al. 2021).<br><br>Data analysis was also performed with Prism v8.0.1 (Graphpad) and Microsoft Excel.

For manuscripts utilizing custom algorithms or software that are central to the research but not yet described in published literature, software must be made available to editors and reviewers. We strongly encourage code deposition in a community repository (e.g. GitHub). See the Nature Research guidelines for submitting code & software for further information.

Data

Policy information about availability of data

All manuscripts must include a data availability statement. This statement should provide the following information, where applicable:

- Accession codes, unique identifiers, or web links for publicly available datasets
- A list of figures that have associated raw data
- A description of any restrictions on data availability

No large scale datasets were generated for this manuscript. Raw data is available from the authors upon request.

Field-specific reporting

Please select the one below that is the best fit for your research. If you are not sure, read the appropriate sections before making your selection.

[x] Life sciences   [ ] Behavioural & social sciences   [ ] Ecological, evolutionary & environmental sciences

For a reference copy of the document with all sections, see nature.com/documents/nr-reporting-summary-flat.pdf

Life sciences study design

All studies must disclose on these points even when the disclosure is negative.

Sample size	For each genotype, a minimum of 2 movies were considered, with each containing between 50-100 imaged nuclei. For each analysis the sample size (number of activated nuclei) is indicated. For nonparametric estimation of the waiting time distribution we have typically used 200 transcription sites resulting in 20 000-50 000 waiting time sample size. The resulting confidence intervals were small enough to allow discrimination between models with 2 states and 3 states. The sample size is large enough to allow robust statistical analysis.
Data exclusions	No data exclusions were made.
Replication	All live embryo data collection was performed on multiple embryos as indicated in text. ChIP-qPCR for Pol II (n=4) and NELF-E (n=2) was followed by qPCR in technical triplicate. qRT-PCR was performed in biological and technical triplicate. smiFISH was performed on multiple embryos as indicated in text. All published replicates were successful.
Randomization	Not applicable. Embryos were selected for suitability for live imaging prior to the time frame of interest. Fixed sample experiments were performed with large pools of embryos of the specified genotype.
Blinding	Not applicable, as quantitative analysis was not manually performed.

Reporting for specific materials, systems and methods

We require information from authors about some types of materials, experimental systems and methods used in many studies. Here, indicate whether each material, system or method listed is relevant to your study. If you are not sure if a list item applies to your research, read the appropriate section before selecting a response.

Materials & experimental systems

n/a	Involved in the study	
[ ]	[x]	Antibodies
[x]	[ ]	Eukaryotic cell lines
[x]	[ ]	Palaeontology and archaeology
[ ]	[x]	Animals and other organisms
[x]	[ ]	Human research participants
[x]	[ ]	Clinical data
[x]	[ ]	Dual use research of concern

Methods

n/a	Involved in the study	
[x]	[ ]	ChIP-seq
[x]	[ ]	Flow cytometry
[x]	[ ]	MRI-based neuroimaging

Antibodies

Antibodies used	rabbit anti-Rpb3 (gift from J. Zeitlinger); rabbit anti-NELF-E (gift from J. Zeitlinger)
Validation	Anti-Rpb3 was generated in the Zeitlinger lab and has been previously validated and published in Shao & Zeitlinger (2017). Anti-NELF-E was generated in the Zeitlinger lab and has been previously published and validated in Shao & Zeitlinger (2017).

Animals and other organisms

Policy information about studies involving animals; ARRIVE guidelines recommended for reporting animal research

Laboratory animals	Multiple transgenic Drosophila melanogaster lines were created by PhiC31-mediated recombination at the VK33 locus in the BL9750 (y1 w1118, PBac VK00033) strain. Embryos for analysis were generated from crosses of MCP-GFP-His2A-RFP virgin females to males of the indicated genotypes for live imaging, and yw virgin females to males of indicated genotype for fixed sample imaging. ChIP-qPCR was performed on 2-4h mixed sex homozygous embryos.
Wild animals	This study did not involve wild animals.
Field-collected samples	This study did not involve field samples.
Ethics oversight	No ethical approval or guidance was required for the use of D. melanogaster.

Note that full information on the approval of the study protocol must also be provided in the manuscript.
